# Multi-drug resistant *Salmonella enterica* serovar Typhi isolates with reduced susceptibility to ciprofloxacin in Kenya

**DOI:** 10.1186/s12866-018-1332-3

**Published:** 2018-11-14

**Authors:** Winnie C. Mutai, Anne W. T. Muigai, Peter Waiyaki, Samuel Kariuki

**Affiliations:** 1 Department of Medical Microbiology, University of Nairobi, Nairobi, Kenya; 2 School of Biological Sciences, Jomo Kenyatta University of Agriculture and Technology, Juja, Kenya; 3 Centre for Microbiology Research, Kenya Medical Research Institute, Nairobi, Kenya

**Keywords:** Multi drug resistance in Kenya, *Salmonella enterica* serotype Typhi, Ciprofloxacin

## Abstract

**Background:**

Typhoid fever remains a public health concern in developing countries especially among the poor who live in informal settlements devoid of proper sanitation and clean water supply. In addition antimicrobial resistance poses a major challenge in management of the disease. This study assessed the antimicrobial susceptibility patterns of *Salmonella enterica* serotype Typhi (*S*. Typhi) isolated from typhoid fever cases (2004–2007).

**Methods:**

A cross sectional study was conducted on 144 archived *S.* Typhi isolates (2004–2007) tested against 11 antimicrobial agents by quality controlled disk diffusion technique. Isolates resistant to ampicillin, chloramphenicol, and cotrimoxazole were considered Multidrug resistant (MDR). Thirty MDR isolates were selected randomly and further tested using minimum inhibitory concentration (MIC) E-test.

**Results:**

Sixteen percent (23/144) of the isolates were susceptible to all the antibiotics tested while 68% were resistant to three or more of the 11 antibiotics tested. The isolates showed a high susceptibility to ceftriaxone (94%) and gentamicin (97%). A high percentage of resistance was observed for the conventional first-line antibiotics; ampicillin (72%), chloramphenicol (72%), and cotrimoxazole (70%). Sixty-nine percent of the isolates (100/144) showed reduced susceptibility to ciprofloxacin. All the 30 (100%) isolates selected for MIC test were susceptible to amoxicillin-clavulanic acid. All except one of the 30 isolates were susceptible to ceftriaxone while majority 21 (70%) recorded an intermediate susceptibility to ciprofloxacin with MIC of 0.12–0.5 μg/mL.

**Conclusion:**

A large proportion of *S*. Typhi isolates were MDR and also showed reduced susceptibility to ciprofloxacin. Fluoroquinolone resistance is emerging and this may pose a challenge in treatment of typhoid in future. There is need for routine surveillance to monitor this phenotype in clinical settings.

## Introduction

Typhoid fever posses a public health threat, recording high morbidity and mortality rates mainly in developing countries [1, 2]. Current published data on typhoid fever shows an annual global estimate of 20·6 million cases and 223 000 deaths. In sub-Saharan Africa, the incidence of typhoid fever is greater than 100 per 100,000 persons per year resulting in 33, 490 deaths accounting for 26% of global typhoid deaths in Africa [3]. Incidence may vary from one country to another due to dynamics in risk factor exposure levels attributed to the disease; some countries like Egypt report low incidence rate (13/100,000–59/100,000 persons annually) [4–6] while others like Kenya have reported high adjusted incidence rate of up to 247 cases per 100,000 persons in an informal settlement [7]. These figures may, however, be overestimated based on the controlled risk factors. The ongoing ‘Typhoid Fever Surveillance in Africa’ study may probably give the accurate estimate [8].

The high incidence rates of typhoid fever have been exacerbated by the emergence of *S.* Typhi strains resistant to antibiotics recommended for treatment. Centers for Disease Control and Prevention (CDC) ranks antibiotic resistant *S.* Typhi as a serious threat that requires frequent monitoring and prevention to reduce the spread of the resistant strains [9]. In the past, first line antibiotics for the treatment of typhoid included chloramphenicol, ampicillin, and trimethoprim-sulphamethoxazole. However, Multidrug resistant (MDR) *S.* Typhi, defined as strains resistant to these first-line antibiotics emerged in the late 1980s [10, 11]. This resulted in the use of fluoroquinolones and third generation cephalosporins as alternatives for treatment of MDR *S.* Typhi cases.

Increased use of fluoroquinolones such as ciprofloxacin for treatment has resulted in the emergence of strains resistant or with reduced susceptibility to this particular antibiotic [12]. Other than the use of these antibiotics in the treatment of human cases, there have been many reports on the use of antibiotics in treatment of animal disease and as growth promoters in food derived from animals which similarly contribute to the occurrence and spread of antibiotic resistant bacteria [13].

Based on the current revised Clinical and Laboratory Standards Institute (CLSI) guidelines it is important to have a clear picture of the situation regarding resistance, especially to ciprofloxacin. This study aimed at determining the antimicrobial susceptibility patterns of archived isolates of *S.* Typhi isolated from blood samples.

## Material and methods

### Study design

This was a cross sectional study where 144 *S.* Typhi archived isolates collected between 2004 and 2007 were analysed.

### Bacterial isolation

The isolates were obtained from blood samples of patients presenting with typhoid fever at Aga Khan University Hospital, Nairobi (AKUH) and Kenyatta National Hospital (KNH), both located in Nairobi, Kenya. The archived isolates were randomly selected and subcultured on MacConkey agar to check for purity. Confirmed *S*. Typhi isolates were stored at − 70 °C on freezing media until analyzed. The study was approved by KEMRI Scientific Steering Committee (SSC No. 1320).

### Antibiotic susceptibility testing

Discrete *S.* Typhi isolates were tested for susceptibility to various antimicrobials agents by quality controlled disk diffusion technique based on the CLSI guidelines [14]. The antibiotics (Oxoid Ltd., Basingstoke, United Kingdom) screened included; ampicillin (10 μg), amoxicillin-clavulanic acid (30 μg), cefuroxime (30 μg), ceftriaxone (30 μg), cefotaxime (30 μg) ciprofloxacin (5 μg), nalidixic acid (10 μg), tetracycline (30 μg), chloramphenicol (30 μg), cotrimoxazole (25 μg) and gentamicin (10 μg). The results were interpreted as sensitive, intermediate or resistant in accordance with CLSI guidelines [14]. *Escherichia coli* ATCC 25922 was used as the control strain.

### Case definition

MDR strains were defined as isolates resistant to ampicillin, chloramphenicol, and cotrimoxazole.

### Determination of minimum inhibitory concentrations

Thirty *S*. Typhi isolates that were resistant to ciprofloxacin, ceftriaxone, cefotaxime, amoxicillin-clavulanic acid, nalidixic acid and chloramphenicol by disc diffusion technique were randomly selected from the 98 MDR isolates for MIC determination. The MICs of the antibiotics were determined by E-test guided by the manufacturer’s instructions (AB Biodisk, Solna, Sweden). The cutoff MIC provided by CLSI guidelines were used to interpret the results. *Escherichia coli* ATCC 25922 was used to control for growth of test strains and potency of the E-test strips [14].

### Data analysis

Data generated from this study was entered on MS excel and analyzed using SPSS version 22. The zone of inhibition by disc diffusion was measured in millimeters and interpreted as susceptible, intermediate or resistant based on the CLSI standards. The MIC results of E-test were also categorized as susceptible, intermediate or resistant. Frequency of multidrug resistance isolates was categorized by year of isolation and phenotypes.

## Results

### Antibiotic susceptibility profile

A total of 144 archived and confirmed *S.* Typhi isolates were analysed, 115 were obtained from Aga Khan University Hospital and 29 from Kenyatta National Hospital. Twenty three isolates were fully susceptible to all the antibiotics tested. Most of the isolates tested recorded a high susceptibility to ceftriaxone (94%), gentamicin (97%), cefotaxime (83%) and amoxicillin-clavulanic acid (81%). A high resistant rate was observed among the first-line antibiotics, ampicillin (72%), chloramphenicol (72%) and cotrimoxazole (70%). Sixty-nine percent of the isolates showed an intermediate susceptibility to ciprofloxacin while 6% were fully resistant. Antibiotic susceptibility test for the 144 isolates is summarized in Table 1.

**Table 1:** Antibiotic susceptibility patterns among the 144 *S.* Typhi isolates

Drug	Susceptible	Intermediate	Resistant
	Number (%)	Number (%)	Number (%)
Amoxycillin-clavulanic acid	117 (81)	8 (6)	19 (13)
Ampicillin	34 (24)	6 (4)	104 (72)
Cefotaxine	120 (83)	16 (11)	8 (6)
Ceftriaxone	136 (94)	3 (2)	5 (4)
Chloramphenicol	39 (27)	1 (1)	104 (72)
Cefuroxime	98 (68)	38 (27)	8 (6)
Ciprofloxacin	35 (24)	100 (69)	9 (6)
Gentamicin	140 (97)	0 (0)	4 (3)
Nalidixic acid	102 (71)	32 (22)	10 (7)
Tetracycline	37 (26)	2 (1)	105 (73)
Cotrimoxazole	41 (29)	2 (1)	101 (70)

### Multi- drug resistant *S.* Typhi

Ninety eight (68%) of the isolates were MDR. The highest MDR phenotype observed were those resistant to four drugs (98.6%). The frequency of MDR isolates seemed to be reducing from 2004 to 2007 and this was reflected in both hospitals as shown in Table 2.

**Table 2:** Distribution of MDR *S.* Typhi

Parameter	Aga-Khan University Hospital *N* = 115 (%)	Kenyatta National Hospital *N* = 29 (%)	Total no. of MDR isolates
No. of MDR *S.* Typhi	75 (65%)	23 (79%)	98 (68%)
Year of isolation			
2004 (*n* = 59)	31 (53%)	17 (29%)	48 (81%)
2005 (*n* = 47)	25 (53%)	4 (9%)	29 (62%)
2006 (*n* = 37)	19 (51%)	2 (5%)	21 (56%)
2007 (n = 1)	0 (0%)	0 (0%)	0 (0%)
No. of resistant antibiotics			
3 (*n* = 8)	3 (37.5%)	0 (0%)	3 (37.5%)
4 (*n* = 69)	58 (84%)	10 (14.5%)	68 (98.6%)
5 (*n* = 22)	10 (45.5%)	10 (45.5%)	20 (91%)
6 (*n* = 4)	3 (75%)	1 (25%)	4 (100%)
7 (*n* = 2)	0 (0%)	1 (50%)	1 (50%)
9 (*n* = 1)	1 (100%)	0 (0%)	1 (100%)
11 (*n* = 1)	0 (0%)	1 (100%)	1 (100%)

### Minimum inhibitory concentration

All the 30 (100%) isolates selected for MIC test were susceptible to amoxycillin-clavulanic acid. About 97% were susceptible to ceftriaxone. Five isolates were resistant to cefotaxime (17%) and fourteen (47%) were resistant to chloramphenicol. Of the nine isolates that were resistant to ciprofloxacin by disc diffusion only four exhibited complete resistance to ciprofloxacin (13%) with a MIC ≥1 μg/mL. The majority (70%) of the isolates tested recorded intermediate susceptibility with MIC of 0.12–0.5 μg/mL. In addition from the 10 isolates resistant to nalidixic acid by disc diffusion, resistance was observed in six isolates (MIC ≥32 μg/mL) while 24 of the isolates tested were fully susceptible (MIC < 16 μg/mL) (Table 3).

**Table 3:** Minimum inhibitory concentrations to antimicrobials

Antibiotic	Susceptible (%)	Intermediate (%)	Resistant (%)
Cefotaxime	23 (77)	2 (6)	5 (17)
Chloramphenicol	16 (53)	–	14 (47)
Ciprofloxacin	5 (17)	21 (70)	4 (13)
Ceftriaxone	29 (97)	1 (3)	0 (0)
Nalidixic acid	24 (80)	–	6 (20)
Amoxycillin-clavulanic acid	30 (100)	0 (0)	0 (0)

## Discussion

Following the analysis of the trends in susceptibility pattern of *S*. Typhi, the results from this study showed that only a small percentage (16%) of the isolates were susceptible to all the eleven drugs tested while 96% were resistant to one or more of the eleven antibiotics tested. Over the years prevalence of MDR *S.* Typhi has been on the increase in Kenya since it was first reported in 1997–1999 where then the prevalence of MDR phenotype was estimated at 50 to 65% [15]. The prevalence has since then been on the increase based on the previous studies that have been conducted. In 2001–2002 a prevalence of 70 to 78% of MDR *S.* Typhi was reported [16] and in 2010 Mengo and colleagues recorded a prevalence of 70% [17]. These figures are consistent with what was reported in this study where 68% of the isolates were MDR (Table 2). This is however in contrast to a study from a tertiary Care Hospital in Coastal Karnataka, India, that reported an MDR proportion of 1.94% of *S*. Typhi isolates from blood samples [18] while other studies in Nepal have recorded no MDR strains [19]. Over the counter prescription, self-medication and unrestricted use of these drugs may have driven the consistent increase in the prevalence of MDR strains [20]. The use of antibiotics such as tetracyclines, sulfonamides and trimethroprime, nitrofurans, aminoglycosides, β-lactams, and quinolones by farmers as growth promoters for livestock production could also be predisposing individuals to resistant pathogens [13, 20].

In our study a high proportion of the isolates were resistant to the conventional first line antibiotics (ampicillin (72%)^,^ cotrimoxazole (70%) and chloramphenicol (72%). Previously, Mengo et al. also recorded high resistance to ampicillin (75%)^,^ cotrimoxazole (73%) and chloramphenicol (74%) in Kenya [17]. Contrary to the current research on antibiogram of *S*. Typhi, the bacterium is showing full sensitivity to these antibiotics since they had not been used for a long time to treatment typhoid fever [21]. A study in Nepal reported an increased susceptibility rate of chloramphenicol, co-trimoxazole, and ampicillin as 98.8, 98.8, and 97.6% respectively [22]. These rates are quite high and show promising reemergence of strains susceptible to these drugs that can then be reconsidered for the treatment of typhoid fever.

Ciprofloxacin has been used as an alternative antibiotic in the treatment of MDR cases. However, with the currently reviewed breakpoints of ciprofloxacin by CLSI, there has been a rather increase of isolates resistant or recording reduced sensitivity to this antibiotic. In this study, MIC results of ciprofloxacin showed that 13% of the isolates were resistant. Reduced susceptibility to ciprofloxacin, poses a serious threat to the treatment failure of typhoid fever, especially in developing countries. Similar findings have been reported in other African countries. In Malawi 100% of all the isolates tested were MDR and 10% were resistant to nalidixic acid. In the Democratic Republic of Congo 30% were MDR of which 15% showed nalidixic acid resistance and decreased susceptibility to ciprofloxacin [23, 24]. Based on a National Typhoid and Paratyphoid Fever Surveillance System (NTPFS) in the US among travelers 69% showed reduced susceptibility to nalidixic acid of which 99% of these were either resistant to ciprofloxacin or showed reduced susceptibility [25]. One study in India recorded a 98% of resistance to nalidixic acid among *S*. Typhi isolates from blood cultures [26] this same study reported an increase in susceptibility of the isolates to ampicillin and cotrimoxazole during the study period (2008–2013). Recommendation to use fluoroquinolones for empirical treatment in place of first line antibiotics may have contributed largely to the emergence of fluoroquinolones resistance.

This study documented 94% of sensitivity to ceftriaxone, this antibiotic may, therefore, be used as an alternative in the treatment of typhoid fever considering its low resistance proportions. Studies in Germany, India, and Nigeria [27–29] have however detected the presence of CTM-X gene group of extended-spectrum β-lactamase resistance (ESBL) that confers resistance to ceftriaxone. With recent reports from different countries reporting resistance to ceftriaxone, routine screening of such isolates is important.

### Limitations of the study

We did not determine the MIC of all MDR isolates and the antibiotics tested and further detect the mutations associated with ciprofloxacin and nalidixic acid resistance that confer fluoroquinolones resistance.

## Conclusion

Results from this study indicate that there is a significant variation in resistance pattern among *S.* Typhi isolates to the different antibiotic agents recommended for treatment. MDR *S.* Typhi strains are still high however continuous monitoring of susceptibility to the initial first line antibiotics is necessary since the MDR strains have lately shown increased susceptibility. In addition, there is an emergence of strains resistant and with intermediate susceptibility to ciprofloxacin. Therefore the use of ciprofloxacin for treatment of typhoid fever needs routine surveillance to prevent further spread of these strains.

## Abbreviations

AKUH: Aga Khan University Hospital
ATCC: American type culture collection
CDC: Centre for disease control and prevention
CLSI: Clinical and laboratory standards institute
ESBL: Extended-spectrum β-lactamase resistance
KEMRI: Kenya medical research institute
KNH: Kenyatta National Hospital
MDR: Multidrug resistant
MDRST: Multidrug resistant *S*.Typhi
MIC: Minimum inhibitory concentrations
NTPFS: National Typhoid and Paratyphoid Fever Surveillance System
WHO: World Health Organization
